# Transformation of coastal wetlands in the Sundarban Delta (1999–2020)

**DOI:** 10.1007/s10661-024-12901-x

**Published:** 2024-07-24

**Authors:** Shouraseni Sen Roy, Tuhin Ghosh, Dishane K. Hewavithana

**Affiliations:** 1 Department of Geography & Sustainable Development, University of Miami, Coral Gables, FL USA; 2 School of Oceanographic Studies, Jadavpur University, Kolkata, India; 3 Institute of Environment, Florida International University, Miami, FL USA

**Keywords:** Mangroves, Pond-aquaculture, LULC, Sundarban, Join count statistics, Hot spots analysis

## Abstract

Spanning across Bangladesh and India, the Sundarban Delta consists of over a thousand islands, the majority of which are protected. These islands are important for the rich biodiversity and unique species found here. However, these islands are also at the forefront of climate change due to the impact of rising sea levels and extreme weather events. Therefore, we analyzed the long-term transformations in the land use land cover (LULC) between 1999 and 2020. We used a variety of geostatistical methods, including optimized hot spots cold spots and join count statistics, to examine the spatial patterns of changes in LULC across the study area. The results of our analysis revealed substantial changes in the spatial patterns of mangroves and pond aquaculture. The changes revealed a distinct north–south demarcation in spatial patterns, in the form of clustering of mangroves in the uninhabited islands located in the south and pond aquaculture clustered in the northern inhabited islands. The loss of area under mangroves was concentrated in the southern edges of the islands, which were most exposed to erosion in the open ocean. Nevertheless, we observed an increase in the area under mangroves in some of the northern riverine islands (17 km^2^). In the case of pond aquaculture, it was mostly concentrated in inhabited islands in the north. Most of the expansions were concentrated in the Indian part of the delta (631 km^2^). It is noteworthy that because of effective conservation measures, there was very limited overlap between mangroves and pond aquaculture, denoting the conversion of agricultural land to pond aquaculture instead of mangroves. Thus, the results of our study revealed the importance of local level conservation policies and anthropogenic activities, such as deforestation and local level disturbance like over-extraction of water and pollution, on the changing patterns of LULC across this unique, fragile ecosystem. Future studies may incorporate a finer resolution time series of LULC changes over time and space to enable more detailed analysis.

## Introduction

**Fig. 1:**
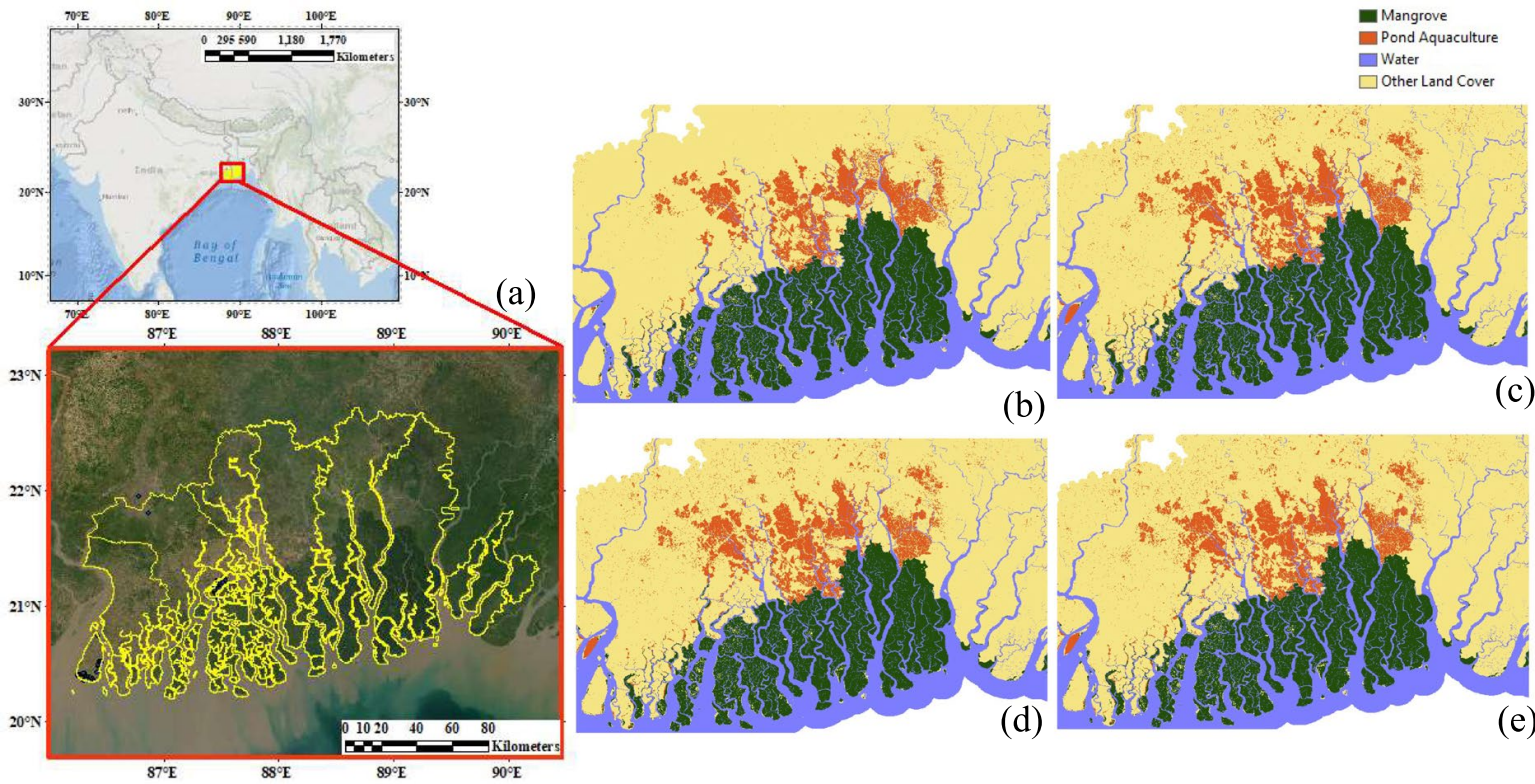
**a** Location of the Sundarban Delta in South Asia. Coastal wetlands: **b** 1999, **c** 2014, **d** 2018, **e** 2020

The effects of projected climate change, combined with non-climatic drivers, are widely expected to cause loss and degradation of much of the world’s low-lying coastal wetlands (IPCC, 2022). In addition to the effect of sea level rise (SLR) and extreme weather, coastal wetlands are experiencing rapid changes in land use land cover (LULC) due to anthropogenic activities. Particularly mangroves, located in the intertidal areas of tropical and subtropical coastal wetlands, are vulnerable to these impacts. A significant proportion of area under mangroves is in south and southeast Asia and play a significant role in providing a variety of ecosystem services such as shore stabilization, reducing the effect of severe weather, ensure water quality and water recharge (Akbar Hossain, et al., 2022; Chowdhury & Hafsa, 2022; Duke et al., 2007; Giri et al., 2015; Pham et al., 2017). Despite its significant positive role in the environment, they are under constant threat of deforestation, with an annual loss of 1–2% (Sardar & Samadder, 2021). In this regard, the area under mangroves in the Sunadarban Delta declined by 1.2% from 1970 to 2000 due to a combination of anthropogenic and natural factors (Giri et al., 2007). Multiple studies have highlighted the local level land use changes, anthropogenic processes, the resulting impacts of tropical cyclones, mangrove destruction, and pond aquaculture in the different parts of the delta (Ellison et al., 2000; Hajra & Ghosh, 2018; Sahana et al., 2020, 2022; Mondal and Das 2023). However, there are no comprehensive studies for the entire delta region identifying the spatial patterns of such changes, which would be useful for informed decision-making, sustainable land management, and the conservation of the delta’s unique ecosystems. Therefore, in the present study, we have examined the spatial patterns of changes in two specific LULC categories: pond aquaculture and mangroves in the Sundarban Delta (Fig. 1a). By using geostatistical analysis techniques, we have identified spatial clusters of the two-land use types and overall LULC changes that have occurred between 1999 and 2020.

## Study area

The Sundarban Delta encompasses one of the largest mangrove forests and is considered one of the most productive natural ecosystems in the world. It is located at the mouth of Ganges, Brahmaputra, and Meghna rivers at the head of Bay of Bengal, covering 10,000 km^2^ across Bangladesh (60% of the total area) and India (40% of the total area) (WWF, 2023) (Fig. 1a). These mangrove forests are well known for their rich diversity of flora (25 true mangroves and 30 mangroves associates) and fauna (tigers, fish, crabs, and a variety of amphibians) (Danda, 2010). This delta is densely populated by human settlements concentrated in its northern half. The main occupation of most of the population is agriculture; they are engaged in monocropping of rice paddies. This occupation has been experiencing a decline in the delta region due to increasing levels of poverty leading to the outmigration of younger population in search of jobs in the neighboring states (Adger et al., 2018; Hajra & Ghosh, 2018), as well as a lower supply of freshwater and saltwater inundation from flooding rendering the land unsuitable for cultivation. Beyond agriculture, other sources of livelihood in the delta include crab and honey collection.

Due to their vulnerable location, these islands are constantly exposed to the impacts of climate change in the form of SLR, tropical cyclones, and saltwater intrusion. Under the projected SLR scenarios, the rich mangrove forests of Sundarban will be able to persist till 2100 (Nicholls et al., 2020). Notwithstanding, the area under mangroves on the Indian side of the delta compared to the Bangladesh side has experienced a steady decline over the last few decades due to SLR, extreme weather events, over-harvesting, pond aquaculture expansion, shrimp and salt farming, regular oil spills, and lack of sustainable adaptative strategies (Ellison et al., 2000; Sahana & Sajjad, 2019; Sahana et al., 2020). At least two islands of the Indian side have disappeared over the last three decades. Additionally, Ghoramara Island lost almost half of its area between 1968 and 2014 (Sarkar et al., 2019). Specifically, some of the villages including Khasimara Char, Lakshmi Narayanpur, Khasimara, and Baishnabpara are already submerged (Ghosh et al., 2003; Jana et al., 2012). This has led to mass migration of the residents of Ghoramara island to nearby islands in the delta, including Sagar. Moreover, the increasing intensity and frequency of tropical cyclones can result in severe irreversible damage to the ecosystem (Nicholls et al., 2020). Since the naming of tropical cyclones in the Bay of Bengal started in 2006, there have been frequent intense named storms including Sidr (November 2007), Rashmi (October 2008), Alia (May 2009), Bulbul (October 2019), Amphan (May 2020), and Yaas (May 2021). Each storm progressively exposes the existing weak infrastructure of the islands. Various resilience measures such as concrete and mud embankments built for protection against storm surges are often destroyed leading to saltwater inundation in agricultural fields. Future model projections reveal a 50% increase in post-monsoonal cyclone formation related to warmer sea surface temperatures resulting from increased greenhouse gas concentration (Danda et al., 2020).

Despite resource limitations and youth outmigration, the area has seen a population boom, driving urbanization and extensive pond aquaculture. Specifically, between 1951 and 2011 the delta’s population grew from 1.15 million to 4.44 million. There is widespread indication of the growth of pond aquaculture in the short term over the next 20 years. This trend in increasing conversion of land to pond aquaculture has been driven by financial considerations (higher income over shorter periods) as well as rising levels of salinity caused by SLR and hydrological mismanagement in the upper delta systems (Rahman et al., 2020). In addition, rising soil salinity due to inundation and SLR renders the land unusable for agriculture. The conversion of agricultural land to pond aquaculture results in a decline in soil quality due to increased level of soil salinity and acidity, which further leads to soil toxicity and mangrove destruction (Ali, 2006; Azad et al., 2009). Thus, it is evident that there are rapid widespread changes in LULC in the delta region due to anthropogenic and natural forces, which have long-term impacts on the sustainability of the islands and their ecosystems in the future.

## Data

Classified aquaculture and coastal habitat data were downloaded from the publicly available aquaculture land cover data produced by Clark Labs (2022). The classified datasets are available for 4 years, 1999, 2014, 2018, and 2020. For 1999, the source images were from Landsat 5, while for the rest of the years, the source images were from Landsat 8. Most of the images were acquired during the dry season, predominantly in April. In some instances, images from multiple dates were classified to get a more complete cloud-free image of various sites (Eastman et al., 2015, 2020). For each image, the raw digital numbers were first converted to top-of-atmosphere reflectances based on the available metadata. In addition, all images were corrected for the solar elevation angle and haze (using Dark Object Subtraction). Finally, the classification of the images was completed using training samples internal to each image, with a 1% dark-object subtraction haze removal procedure used (Chavez, 1996). The spatial resolution of the final classified datasets was calculated at 15 m by merging the 15 m panchromatic band information from bands 3 (blue), 4 (green), and 5 (red). The original spatial resolution of 30 m was reduced to 15 m to correctly detect the pond aquaculture land cover class by using a modified version of the Generalized Intensity-Hue-Saturation (GIHS) pan merge algorithm (Tu et al., 2001). The relationship between the Operational Land Imager (OLI) and the panchromatic bands was established using multiple regression; the resulting coefficients effectively removed the color distortion that usually occurs from this technique (Eastman et al., 2015). Other bands used in the classification analysis included OLI bands 5–7. These bands were upscaled to match the finer spatial resolution of 15 m by using the nearest neighbor and bilinear resampling techniques. Thus, a strong correspondence between the original band and its 15 m equivalent was ensured. Further information about the creation of this dataset is available in Eastman et al. (2015).

The coastal zone was delineated at 10 km on either side of the coastline. Regardless, in some cases, the zone was extended to include marine areas ≤ 30 m based on the GEBCO (2010) bathymetry and land areas ≤ 5 m as defined by the SRTM (2009) elevation data. The maximum extension inland included 60 km, to limit the areas where there was more likelihood of brackish water for pond aquaculture to be used for shrimp aquaculture (Eastman et al., 2015). The classification procedure for most classes was the Mahalanobis Typicality Classifier, the output from which is in the form of typicality probabilities (ranging from 0 to 1, with 1 indicating perfect membership to the class) for each class included in the training process (Foody et al., 1992). Further detailed information about accuracy assessment for the different LULC categories is available in Eastman et al. (2018). These processed classified datasets consisted of four LULC categories: pond aquaculture, mangroves, water, and other land cover. Pond aquaculture included all brackish pond aquaculture. Other land covers included any non-mangrove wetland, fresh, or brackish, that occurred within the defined coastal zone (Eastman et al., 2020).

## Methods

Considering the expansive reach of the Sundarban Delta across India and Bangladesh, datasets for coastal wetlands spanning the years 1999, 2014, 2018, and 2020 were acquired for both nations. The datasets for each year were clipped to the spatial extent of the Sundarban Delta islands and mosaicked to combine them into one image. The final images used for the analysis included four images, one for each year 1999, 2014, 2018, and 2020 (Fig. 1b–e). We next converted the raster datasets to vector format to conduct a detailed local level spatial analysis. Based on the available LULC categories, we limited our analysis to changes in mangroves and pond aquaculture.

**Fig. 2:**
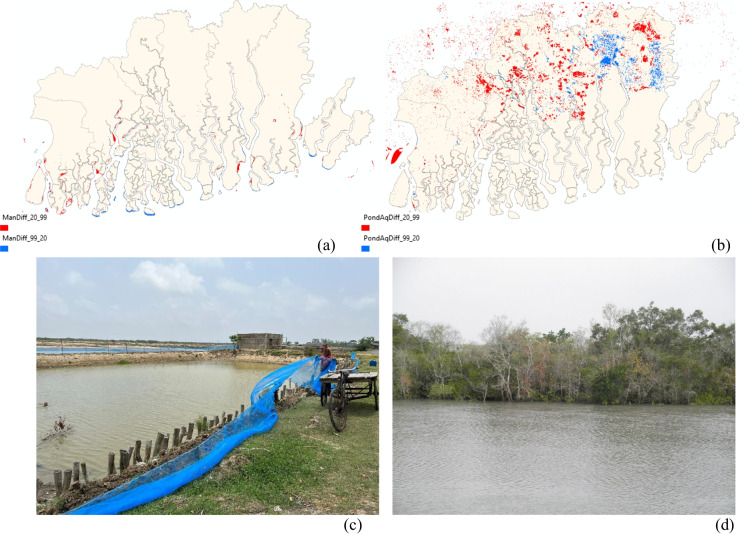
Locations of gains and losses in 1999 vs 2020: **a** mangroves and **b** pond aquaculture. Red color denotes an increase from 1999 to 2020 and blue color denotes a decreased from 1999 to 2020. **c** Installation of pond aquaculture in Sagar Island. **d** Mangroves in Gosaba (photos by authors)

**Fig. 3:**
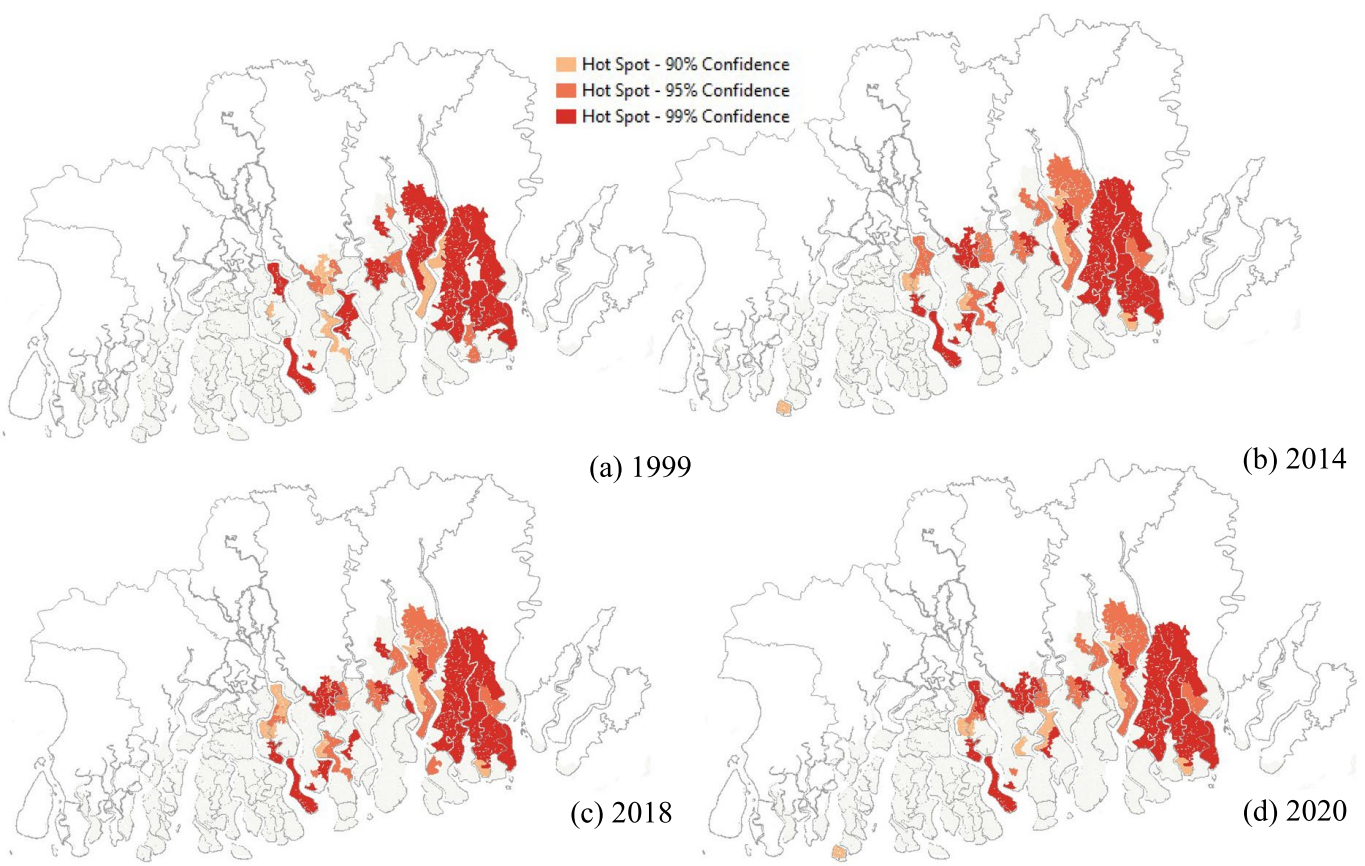
Results of optimized hot spots cold spots analysis of spatial clustering of mangrove parcel size. **a** 1999. **b** 2014. **c** 2018. **d** 2020

**Fig. 4:**
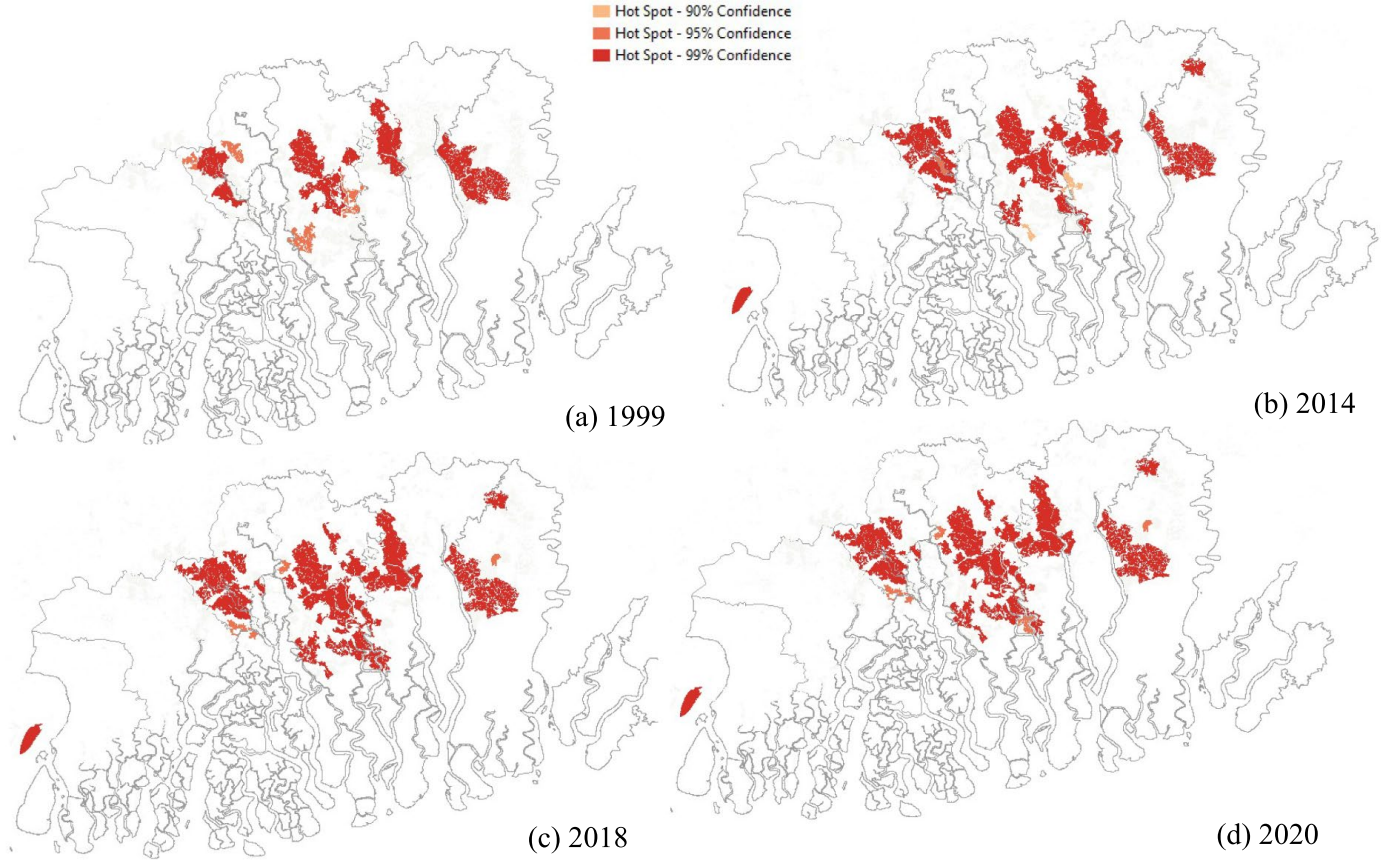
Results of optimized hot spots cold spots analysis of spatial clustering of pond aquaculture parcel size. **a** 1999. **b** 2014. **c** 2018. **d** 2020

We first determined the local level changes in the spatial extent of mangroves and pond aquaculture between 1999 and 2020, by calculating the differences at the local level (Fig. 2). This enabled us to highlight areas of losses and gains during the study period. We next analyzed the spatial clustering of mangroves and pond aquaculture based on fragment size by utilizing optimized hot spots cold spots (OHSCS) analysis. The main objective of this technique is to produce optimal results in the form of statistically significant spatial clusters of high values (hot spots) and low values (cold spots), by evaluating the characteristics of the input features. The automatic aggregation of the features is scale-sensitive and corrected for both multiple testing and spatial dependence (ESRI, 2023). The result of the analysis creates a new output feature class, with induvial *z*-scores, *p*-values, confidence level bin (Gi_Bin), and number of neighbors for each feature in the dataset (Bagwell et al., 2024). The analysis field used in this method included fragment size. The spatial patterns of the results of OHSCS for fragment size of mangroves and pond aquaculture for all four time periods, 1999, 2014, 2018, and 2020 are shown in Figs. 3 and 4.

**Fig. 5:**
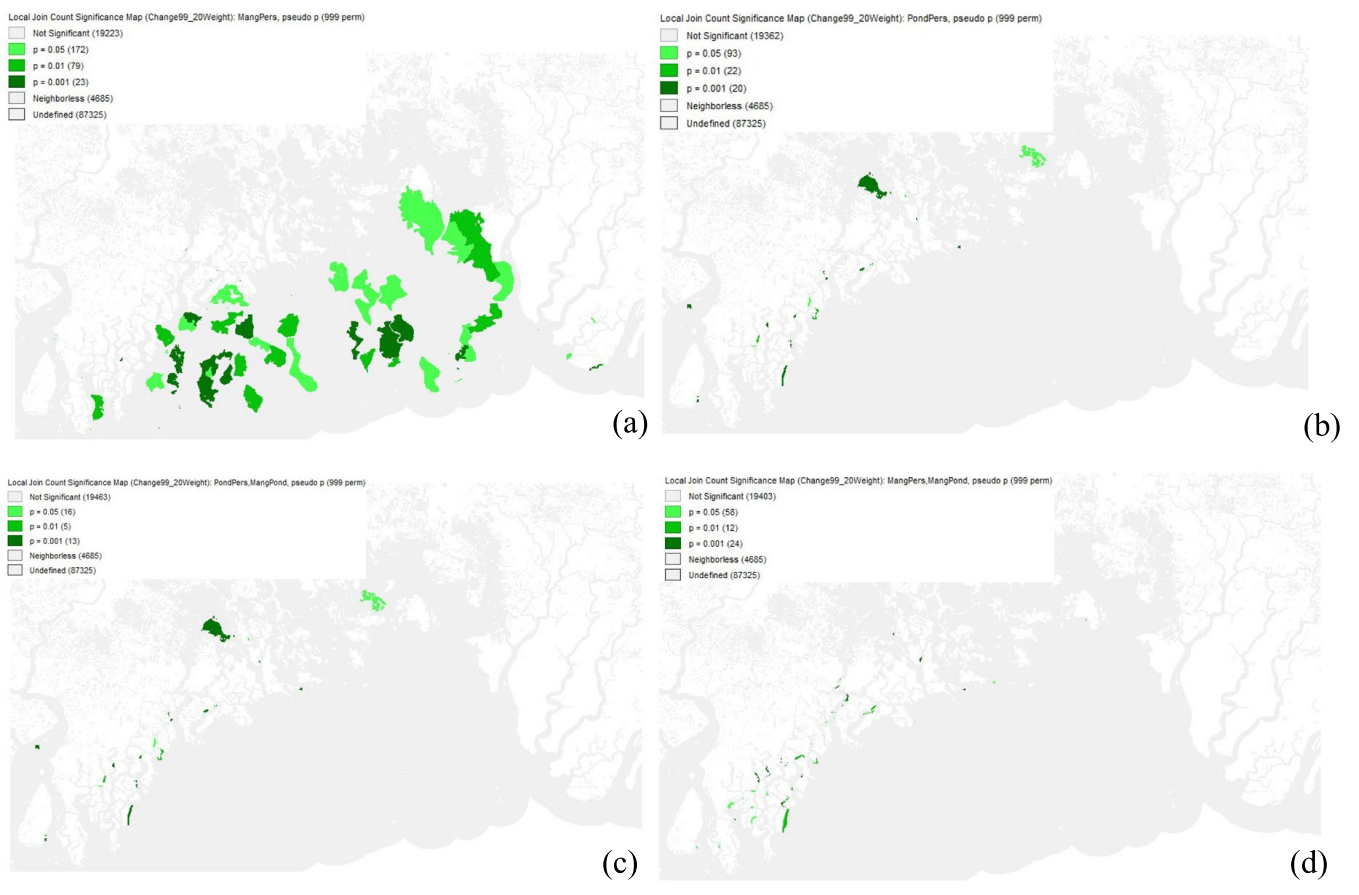
Results of spatial clustering analysis from 1999 to 2020 local joint count analysis: **a** mangroves persistence and **b** pond persistence and bivariate joint count statistics: **c** pond persistence and mangrove to pond and **d** mangrove persistence and mangrove to pond

We next analyzed the spatial clustering of the change in different LULC categories between 1999 and 2020, by using local join count statistics useful for discrete data. The variables analyzed included pond aquaculture to water or mangroves, pond aquaculture persistence, mangroves to pond aquaculture or water, and mangroves persistence. For each of the variables, we utilized local join count statistics to identify the local level spatial autocorrelation. This method consists of counting features that correspond to occurrences of value pairs at neighboring locations, based on a binary variable calculated for each variable separately. The three cases are joins of 1 − 1 (where neighboring features are the selected variable, such as mangroves), 0 − 0 (where both neighboring features are not the selected variable being analyzed), and 0 − 1 (when one of the features is the selected variable but the neighboring feature is not). The first two cases are examples of positive spatial autocorrelation, while the third one is negative spatial autocorrelation. Furthermore, the first case is also indicative of hot spots for the selected variable, which is shown in Fig. 5a and b. This analysis was also conducted for the other time periods, but the results of the maximum change for the longest time periods were presented in this study to highlight the changes across space over time.

Finally, we also used bivariate local join count statistics to detect the co-location of any two selected variables form of spatial relationship between two different variables calculated, with the general form of the equation below:

$$BJ{C}_{i}={x}_{i}\left(1-{z}_{i}\right)\sum_{j}{w}_{ij}{z}_{j}\left(1-{x}_{j}\right)$$

where *wij* is unstandardized (binary) spatial weights, and* x* and *z* are two locations under consideration. Further information about the methods described above is available from Anselin and Li (2019). The results of the analyses are shown in Fig. 5c and d.

## Results and discussion

### Spatial patterns of gains and losses

We observed a distinct separation between the spatial clustering of mangroves and pond aquaculture. Figure 1b–e illustrate these spatial patterns of coastal wetlands, including mangroves, pond aquaculture, and water bodies, spanning from 1999 to 2020. In general, there is a north–south divide with a greater proportion of mangroves located in the southern islands and pond aquaculture in the northern islands. This is indicative of saltwater tolerant mangroves thriving in the saltwater, while the concentration of pond aquaculture in the riverine part of the delta. The clustering of saltwater-tolerant mangroves predominantly in the less human-disturbed southern islands suggests their adaptability to saltwater conditions. In contrast, pond aquaculture expansion is more pronounced in the northern part of the study area, aligning with regions of higher human habitation. The gradual changes in the spatial patterns of the coastal wetlands are distinct through 1999 to 2020, with greater spatial spread of pond aquaculture in the northern part of the delta. Specifically, between 1999 and 2020, the area under pond aquaculture increased by 631 km^2^, compared to mangroves that increased by only 17 km^2^. The local level spatial patterns of gains and losses in the area under mangroves between 1999 and 2020 revealed the concentration of losses in the southwestern edge of the islands, where they are more exposed to open ocean currents (Fig. 2a). On the other hand, the areas that experienced gains in mangroves were in the northern part of the delta, particularly in riverine areas. Furthermore, all the losses and gains in mangroves were expectedly located on the edges of the islands.

Previous studies have documented the varying areas under mangroves over time, including a 1.4% increase between 1970 and 1990, followed by a 2.5% decrease between 1990 and 2000 (Jayappa et al., 2006). However, the area under mangroves in the Indian Sundarban decreased from 22.9% in 1975 to 19.8% in 2020 (Sahana et al., 2022). The overall increase in mangroves in the delta is a result of active measures taken by the local government and nongovernmental organizations toward the protection of mangroves by regrowth, plantation, and aggradations across the different islands. On the other hand, the loss of mangroves is mainly caused by illegal deforestation, expansion of human settlements, agricultural land, and pond aquaculture (Debnath et al., 2024). In the case of the spatial patterns of pond aquaculture, most of the increases were in the western and northern part of study area, which also overlaps with the inhabited islands. Most of the areas that showed loss of pond aquaculture land use were distributed across the eastern islands, with a few parcels scattered among the western islands.

The role of mangroves in building resilience to SLR is critical because mangroves produce organic carbon primarily in the form of litterfall for surface sediments or deposited as peats (Duarte et al., 2005). This sediment deposition can be enhanced in robust aerial root structures of some mangrove species because of slowing water velocities and settling sediment onto coastal soils (Gedan et al., 2011; Krauss et al., 2003) (Fig. 2c). Furthermore, rapidly generating mangrove roots can grow into newly accumulated sediment, resulting in increased surface elevation (Lovelock et al., 2011). On the other hand, maladaptation from the expansion of large-scale high-intensity brackish shrimp cultivation in Bangladesh has resulted in increased soil salinity, acidity, and degradation of soil quality, which can also cause soil toxicity and ultimately destruction of mangroves (Ali, 2006; Azad et al., 2009). The expansion of pond aquaculture conforms with the findings of previous studies, which revealed a 2–3% annual increase over the last two decades (DasGupta et al., 2019a, 2019b; Giri et al. 2022) (Fig. 2d).

### Spatial clustering of parcel size

Given the distinct dichotomy between the spatial distribution of mangroves and pond aquaculture, we next analyzed spatial patterns of parcel size. The average parcel size between 1999 and 2020 for mangroves declined from 2.29 to 2.27 km^2^ in 2020, while it declined from 0.14 to 0.05 km^2^ for pond aquaculture. However, there is widespread evidence of the role of area under mangroves in the stabilization of island areas and elevations in low-lying coastal areas (Krauss et al., 2017; Zhai et al., 2019). These forests also act as a natural protection against natural hazards such as tropical cyclones along the riverbanks and inland areas. Therefore, it is important to analyze the spatial clustering of parcel size to determine the vulnerability of the islands to erosion and subsidence using OHSCS analysis.

The results of OHSCS analysis revealed significant clustering of the larger mangrove parcel size in the eastern part of the delta (Fig. 3). It is also noteworthy that the islands located in the southern edge of the delta also consist of smaller islands, which along with the greater exposure to the ocean waters, make them more vulnerable to mangrove decline. The western part of the delta consists of smaller parcel size, which also makes them more vulnerable to erosion and loss of mangroves. The smaller parcel size of mangrove forests in the Indian Suandarban is in conformity with findings from earlier studies (Sahana et al., 2015).

According to Quader et al. (2017), the degradation of mangroves is caused by a combination of stressors including anthropogenic activities in the form of deforestation and pond aquaculture, increased salinity, and natural hazards in the form of tropical cyclones. The smaller fragment size of the mangrove forests in the western part of the Sundarban is indicative of higher rates of fragmentation resulting in habitat and biodiversity loss. The importance of larger parcel size for species richness and abundance is critical for many of the tropical species who depend on each other for their survival. In addition, higher rates of deforestation leading to greater fragmentation can also alter the microclimates and cause species endangerment. This is particularly critical for the southern islands, which are more exposed in the open ocean and first in the path of destructive tropical cyclones (Sen Roy & Ghosh, 2024).

In the case of pond aquaculture, the larger parcels were expectedly located in the northern islands, which have more inhabitants (Fig. 4), except Nayachar island in the southwest. Specifically, Nayachar island, which is a fishing island, has been planned for solar power generation and pond aquaculture due to its eco-fragile zonation and sensitive soil condition (Majumdar et al., 2012; Times of India, 2022). It was converted to pond aquaculture from 2014 onward. Throughout the rest of the delta, between 1999 and 2020, the clustering of number of larger parcels being used for pond aquaculture expanded in the northwest and southward in the central islands (Fig. 4). Specifically, the number of significantly clustered parcels increased from 123 parcels (average area of 8.4 km^2^) in 1999 to 152 parcels (average area of 10.89 km^2^) in 2020. Overall, there is an increase in larger parcels being converted to pond aquaculture in the northwestern and central part of the Sundarban, which is more protected from the open ocean and subsequent erosion.

Aquaculture plays an increasingly important role in the local economy and livelihoods of inhabitants in this region and is rapidly expanding due to its quick economic benefits. Given the unique location of the islands, both fresh and brackish water aquaculture are practiced here. Freshwater aquaculture is located more in the northern islands, where the water is taken from the river channels while brackish water aquaculture is located more in the southern islands where the water salinity is higher. The expansion of pond aquaculture in the Indian Sundarban Delta has led to a steep increase in the production of fisheries.

### Spatial clustering of different LULC categories

It is evident from the above findings that there have been distinct changes in the spatial patterns of LULC in the Sundarban Delta from 1999 to 2020. Therefore, in this section, we examined the spatial clustering of persistence and conversion between the two predominant LULC categories: mangroves and pond aquaculture, from 1999 to 2020. The specific categories analyzed included mangroves and pond aquaculture persistence, mangroves to pond aquaculture, and vice versa (Fig. 5). The spatial distribution of mangroves and pond aquaculture persistence showed a distinct spatial dichotomy with significant clustering of pond aquaculture persistence (135 polygons) in the north and mangroves persistence (274 polygons) in the southern part of the delta (Fig. 5a, b). However, it is noteworthy that mangrove persistence in the south did not extend to the southernmost islands on edge, which is in conformity with the loss of mangroves observed in the southernmost islands mentioned above. On the contrary, the spatial clustering of areas experiencing conversion, consisting of ponds to mangroves and mangroves to ponds, revealed no clear spatial clustering, which is indicative of the limited overlap between them. The results of co-clustering analysis of the different LULC categories revealed significant clustering in the western part of the study area for pond persistence and mangroves to ponds and mangrove persistence and mangroves to ponds (Fig. 5c, d).

The conversion of mangroves to ponds occurred mainly along the edges of the persistent clusters of mangroves and areas located closer to inhabited islands, providing easier access to anthropogenic activities. The spatial patterns of long-term trends in LULC in the Sundarban delta indicate the conversion of agricultural land to pond aquaculture started after 1990 and accelerated after Cyclone Aila in 2009 (Sahana et al., 2022). This unplanned rapid expansion of pond aquaculture is replacing agricultural land and, ultimately, has negative ecological consequences (Hossain et al. 2013; Hossain and Bhuiyan 2016; Paul & Røskaft, 2013).

According to Ahmad et al. (2010), women in the islands are mostly involved with shrimp larvae collection, which provides them with important informal sources of livelihood. Conversely, it can result in long-term negative impacts on the ecosystem services and aquatic ecology (Azad et al., 2009; Hoq, 2007). There are also concerns about the actual economic benefits of pond aquaculture for the local population due to the limited outflow of profits and its role in lowering food insecurity in the region (Rahman et al., 2020; Swapan & Gavin, 2011). It has been noted that most of the land conversion to pond aquaculture occurred from agricultural land because of higher economic return and market demand and lower agricultural productivity resulting from saltwater inundation (DasGupta et al., 2019a, 2019b; Dubey et al., 2017; Giri et al., 2022). Moreover, the effect of the saltwater intrusion has been observed in coastal areas of India (Bhadra et al., 2020; Sen Roy et al., 2022) and Bangladesh (Mahmuduzzaman, et al., 2014). Nonetheless, the local level trends in saltwater intrusion in this region need further investigation, in view of the rapidly expanding area under pond aquaculture, consisting of both freshwater and brackish water. More recently, there has been a lot of discussion about the long-term negative socio-ecological effects of the rapid expansion of pond aquaculture expansion in Sadeshkhali, one of the northern islands on the Indian side of the delta (Ghosh & Modak, 2024).

## Conclusions

The Sundarban Delta spread across Bangladesh and India is home to one of the largest halophytic mangroves in the world with a rich biodiversity juxtaposed with increasing population pressure at the forefront of the impacts of climate change. Comprised of multiple islands of different sizes, there have been significant changes in the LULC during the recent past. Therefore, in the present study, we examined the local level spatial patterns of changes in two main types of LULC, including mangroves and pond aquaculture in the coastal wetlands of the Sundarban Delta. In this study, we utilized a publicly available dataset developed by Clark Labs for four years, 1999, 2014, 2018, and 2020, for pond aquaculture and coastal mangrove habitats. The analyses were conducted using geostatistical analysis techniques to identify the local level spatial clustering of the various LULC categories and changes over time. The main findings of our study are summarized below:

- There was a distinct spatial dichotomy between the location of mangroves in the southern islands, which consist of protected forests and pond aquaculture overlapping with inhabited islands in the north.
- The spatial patterns of change of area under various mangroves and pond aquaculture between 1999 and 2020 revealed noticeably different spatial clusters across the study area. The areas where mangrove cover increased were primarily on the northern edges of certain islands in the interior, whereas the losses in mangrove area were predominantly observed along the southern edges of the islands, facing the open ocean. The spatial patterns of pond aquaculture gains were more prominent and widespread in the western (Indian) part, while the losses were relatively smaller spatial extent located in the eastern (Bangladesh) part.
- The detrimental role of fragmentation on the long-term health of ecosystems has been widely validated. Our analysis of spatial patterns of mangroves parcels based on size revealed consistently significant clustering in the eastern part of the delta for all four years. In the case of pond aquaculture, the spatial clustering of larger parcels was expectedly located in the northern islands with a trend of spreading southward in the later years. The spread of pond aquaculture southward has been noted in previous studies due to the lucrative livelihood options for the residents.
- The assessment of changes in the two LULC categories revealed limited overlap between mangroves and pond aquaculture. This denotes that most of the conversion of land to pond aquaculture occurred from agricultural land. Some of the reasons for this trend are saltwater inundation during tropical cyclones, declining agricultural yield, and higher short-term economic returns from pond aquaculture.

It is evident from the above analysis that there are significant spatial patterns in the LULC changes across the delta. The role of anthropogenic activities and local policies drive the patterns of these changes, such as protected forests and conversion of land use from agriculture to pond aquaculture. The fragmentation or disturbance of natural mangroves, specifically those located closer to inhabited islands, will result in the shrinking of natural mangroves, which will take a long time to restore. The loss of mangroves can not only affect the rich biodiversity of the delta, but also accelerate the effects of SLR through erosion and subsidence (Mondal et al., 2021). Therefore, the protection and restoration of mangroves including planting and regrowth need more systematic planning for successful conservation. There is a critical need for a more comprehensive evaluation for a more sustainable and resilient form of pond aquaculture, that can benefit both the local communities and protect the ecosystem in the long-term. Finally, the results of this study can inform and applied to other low lying coastal regions experiencing impacts of climate change.

## Data availability statement

Datasets used in this study are available from https://clarklabs.org/wp-content/uploads/2019/01/Aquaculture-and-Coastal-Habitats-Report-No1.docx.pdf.
